# Alexithymia as a predictor of treatment resistance in Depressive Disorders: neurocognitive profile and therapeutic implications

**DOI:** 10.1192/j.eurpsy.2026.11396

**Published:** 2026-07-31

**Authors:** S. Sorokin, M. Popov

**Affiliations:** Department for the Study of Endogenous Mental Disorders and Affective States, Mental health research center, Moscow, Russian Federation

## Abstract

**Introduction:**

Alexithymia, characterized by difficulties in identifying and describing feelings, represents a significant predictor of treatment resistance in major depressive disorder (MDD). Understanding its neurocognitive underpinnings is crucial for developing targeted interventions for treatment-resistant depression (TRD).

**Objectives:**

This study aims to identify specific neurocognitive correlates of alexithymia in patients with treatment-resistant depression and to develop evidence-based recommendations for therapeutic interventions targeting these cognitive deficits.

**Methods:**

45 patients with MDD (ICD-10 F32, F33) and inadequate response to ≥2 antidepressant trials were stratified using the Toronto Alexithymia Scale (TAS-20) into high-alexithymia (HA, n=22) and low-alexithymia (LA, n=23) groups. Assessment included: 1) Psychometric evaluation using HDRS-17 and TAS-20; 2) Neuropsychological battery: Wisconsin Card Sorting Test (WCST) for executive functions, Emotion Recognition Task (ERT) for facial affect identification, modified Stroop Task with emotional words for attentional bias, and verbal fluency tasks.

**Results:**

The HA group demonstrated significantly more perseverative errors on WCST (p<0.01) and lower ERT accuracy for neutral and sad expressions (p<0.05). Strong negative correlations emerged between TAS-20 scores and WCST categories completed (r=-0.62, p<0.01) and ERT total accuracy (r=-0.58, p<0.01). HA patients showed significantly longer reaction times on emotional Stroop trials (p<0.05), indicating specific attentional biases in emotional processing.

**Conclusions:**

The findings demonstrate that alexithymia in treatment-resistant depression is associated with distinct neurocognitive deficits across executive functioning, emotion recognition, and attentional processing domains. These results support the development of targeted interventions including executive function training using modified WCST paradigms, emotion differentiation therapy through graded exposure to emotional stimuli, metacognitive strategies for enhancing emotional verbalization, and attention modification techniques to reduce cognitive biases. Implementation of these neurocognitively-informed approaches alongside standard depression treatment offers promising avenues for addressing alexithymia-mediated treatment resistance and improving clinical outcomes in this challenging patient population.

**Disclosure of Interest:**

None Declared

